# Attitudes and Perceptions about Clinical Guidelines: A Qualitative Study with Spanish Physicians

**DOI:** 10.1371/journal.pone.0086065

**Published:** 2014-02-05

**Authors:** Ivan Solà, José Miguel Carrasco, Petra Díaz del Campo, Javier Gracia, Carola Orrego, Flora Martínez, Anna Kotzeva, Imma Guillamón, Enrique Calderón, Idoia de Gaminde, Arturo Louro, Rafael Rotaeche, Flavia Salcedo, Paola Velázquez, Pablo Alonso-Coello

**Affiliations:** 1 Iberoamerican Cochrane Centre - Clinical Epidemiology and Public Health Department, Biomedical Research Institute Sant Pau (IIB Sant Pau), Barcelona, Spain; 2 University of Navarra, Institute for Culture and Society, Pamplona, Spain; 3 Agency for Health Quality and Assessment of Catalonia (AQuAS), Barcelona, Spain; 4 CIBER de Epidemiología y Salud Pública (CIBERESP), Barcelona, Spain; 5 Scientific Committee of Guíasalud - National Clinical Practice Guideline Programme of the NHS, Madrid, Spain; 6 Instituto de Biomedicina de Sevilla, Hospital Universitario Virgen del Rocío/CSIC/Universidad de Sevilla, Seville, Spain; 7 Servicio de Docencia y Desarrollo Sanitarios, Departamento de Salud Gobierno de Navarra, Spain; 8 Centro de Saúde de Cambre. Xerencia de Atención Primaria A Coruña – SERGAS –, A Coruña, Spain; 9 General Secretariat of Public Health, Social Inclusion and Quality of Life, Andalusian Regional Ministry of Health and Social Well-being, Seville, Spain; 10 Avedis Donabedian Research Institute (FAD), Barcelona, Spain; 11 Grupo Kronikgune sobre gestión del conocimiento, Centro de Salud de Alza, Donostia-San Sebastián, Spain; 12 Instituto Aragonés de Ciencias de la Salud, Zaragoza, Spain; 13 Universitat Autònoma de Barcelona, Barcelona, Spain; 14 Red de investigación en servicios de salud en enfermedades crónicas (REDISSEC), Barcelona, Spain; University of Louisville, United States of America

## Abstract

**Background:**

Clinical guidelines (CGs) are popular for healthcare decision making but their acceptability and use by healthcare providers is influenced by numerous factors. Some of these factors are professional-related, such as knowledge and perceptions of and attitudes toward CGs in general. The aim of our study was to evaluate attitudes and perceptions of Spanish physicians towards CGs.

**Methods:**

We coordinated six discussion groups with a total of 46 physicians. The participants were drawn from 12 medical specialties from both specialized and primary care. We recorded the sessions and transcribed the content verbatim. We analyzed the data using an approach based on the grounded theory.

**Results:**

We identified two main constructs that defined the physicians' perceptions towards guidelines: knowledge and usefulness. “Knowledge” defined the theoretical meanings of guidelines, while “Usefulness” referred to the pragmatic approach to guidelines. These constructs were interrelated through a series of categories such as confidence, usability, accessibility, dissemination and formats.

**Conclusions:**

In our study, the constructs that impacted most on physician's attitudes to clinical guidelines were knowledge and usefulness. The tension between the theoretical and the pragmatic constructs determined the attitudes and how physicians use guidelines. Groups developing guidelines should ask relevant clinical questions and develop implementable and context specific recommendations. Developers should be explicit and consistent in the development and presentation of recommendations.

## Background

Evidence-based medicine (EBM) has driven the use of tools for the critical synthesis of the literature, including systematic reviews and, more significantly, clinical guidelines (CGs). The former were recently defined as “statements that include recommendations intended to optimize patient care that are informed by a systematic review of evidence and an assessment of the benefits and harms of alternative care options” [1]. Noticeably, the definition highlights the necessity to support recommendations based on the results of rigorous systematic reviews to address specific research questions that minimize bias by using explicit methods [1], [2]. The last few years have seen some significant changes in the methods concerning the development of CGs, and methodological working groups such as GRADE have concentrated on providing transparent, systematic, methodologically-sophisticated guidance for each step in the formulation of recommendations [3].

Although concern has been raised over the trustworthiness of guidelines, several international initiatives have been put forward in order to develop credible guidelines. These endeavors, amongst other things, have targeted the vexing problem of guidelines and conflicts of interest, balancing the pros of having input from experts and assuring that conflicts of interest do not influence recommendations [4], [5]. At the same time, methodologists continue work to develop frameworks and tools to facilitate the development and dynamic updating of trustworthy guidelines [6].

Health professionals' attitudes and expectations concerning EBM and CGs share some similarities. These include aspects such as a generally positive attitude toward bringing rigor to clinical practice, the need to improve their skills and the difficulties in the implementation of the evidence in real life [7], [8]. In general, evidence shows physicians are highly satisfied with guidelines [9], [10]. In a Canadian survey, physicians considered CGs a useful educational tool to guide their practice and improve quality of care [11]. Nevertheless, the practical impact of these documents was of concern, particularly as to their application in individual patients, their role in resource saving, and their potential for a possible increase of lawsuits [11]. A meta-synthesis of qualitative studies recently showed that although GPs have a positive attitude to CGs, they do not follow them for a number of reasons, mostly related to the lack of applicability to the particular patient's needs [12]. The analysis showed that physicians' reasons to adhere to CGs or not differed depending on whether the guideline was prescriptive, that is, whether it promoted a certain type of behavior or treatment, or proscriptive, that is, if it discouraged certain treatments or behaviors [12].

In Spain, and despite the fact that CGs play a central role in the strategies to improve decision-making, several studies show that the implementation of CGs has certain drawbacks, such as the limited systematization and coordination of resources, the proliferation of guidelines that contrast with suboptimal updating, the redundancy in the subjects addressed, and biases in their methodology [13]. Other experiences have also stressed the perceived need of transparency through the process of development and implementation of guidelines [14].

Another factor that may imply a barrier to the use of CGs is the existence of multiple systems for the formulation of recommendations, which sometimes have important limitations [15]. This hinders understanding and communication. The lack of transparency in the criteria applied in evaluating evidence and grading of the recommendations, the unclear estimation of a benefit/risk ratio, and the absence of the grading of the relative importance of outcomes of interest are aspects that limit the credibility of recommendations and, hence, may limit implementation. There is little research evaluating how these systems may influence the perception among health professionals. Some authors have argued that the typical classification of the recommendations using letters, symbols or numbers has been scarcely evaluated [16], [17]. Finally, empirical evidence suggests that their wording could improve their implementation [18], [19]. For all the above-mentioned arguments, we undertook this study to explore knowledge, perceptions and attitudes of health care professionals towards CGs, grading systems, and factors contributing to their understanding, acceptance, and use.

## Methods

Qualitative research based on discussion groups. We reported the complete details of the study protocol elsewhere [20]. This qualitative research method allows comprehensive approximations to the constitutive processes of reality and the perceptions of specific groups, an in-depth analysis of their subjectivity, and an understanding of participant's experiences and beliefs [21]–[23]. This study is part of a mixed methods research project which includes a nationwide survey among health professionals.

First, we organized six discussion groups in different geographical areas to reflect the regional differences in the organization of healthcare services in Spain. Enrolment of the 46 participating physicians from twelve medical specialties was carried out considering a purposive sample [24], [25]. Group composition was based on the level of care in which physicians provided professional assistance (specialized or primary care) and by the knowledge declared in evidence-based medicine. In the case of physicians who provide services in specialized care, a greater representativeness of clinical rather than surgical profiles was sought. Furthermore, to guarantee basic group homogeneity and intergroup heterogeneity, an appropriate age and sex distribution was considered. The first contact with informants was by letter or by telephone. After consent was granted, participants were reminded by SMS of the day when the discussion groups would take place. Table 1 outlines the main characteristics from participants in the discussion groups. The study has been approved by the Ethics Board of the Hospital de la Santa Creu i Sant Pau, Barcelona, Spain. Participants provided written informed consent to participate in this study.

**Table 1:** Characteristics of the participants in the discussion groups.

Location	Number of participants	Age	Sex		Specialties	Years in clinical practice	Experience with CG		Experience in epidemiology	
			Female	Male		Mean	Yes	No	Yes	No
Barcelona	8	26–36 years: 2	6	2	- Anesthesiology	18	1	7	0	8
		37–47 years 5			- Internal Medicine					
		>65 years: 1			- Pneumology					
					- Surgery					
					- Pediatrics					
Barcelona	7	26–36 years: 3	6	1	- Community/Family Doctor	13	1	6	1	6
		37–47 years 3								
		48–58 years 1								
Madrid	7	37–47 years: 6	4	3	- Community/Family doctor	18	6	1	7	0
		48–58 years 1			- Pediatrics					
Seville	9	26–36 years: 2	3	6	- Pediatrics	22	2	7	1	8
		37–47 years: 2			- Internal Medicine					
		48–58 years 4			- Community/Family doctor					
		58–65 years: 1			- Nursing					
Zaragoza	7	37–47 years:3	5	2	- Psychiatry	25	7	0	3	4
		48–58 years: 2			- Radiotherapy					
		59–65 years: 1			- Nephrology					
		>65 years: 1			- Pediatrics					
					- Preventive Medicine and Public Health					
Zaragoza	8	37–47 years:3	3	4	- Anesthesiology	23	0	7	2	5
		48–58 years 4			- Gastroenterology					
		59–65 years 1			- Surgery					
					- Pathology					
					- Emergency Medicine					
					- Psychiatry					

We designed a protocol to guide the moderation and the role of the observers, and to homogenize the process between groups. Similarly, we prepared a background document with general topics to be addressed in the group interviews, and with suggestions of both open and concrete questions that could be of assistance or guidance for moderators. To explore participants' perception regarding the different systems of classification and grading of the evidence, we prepared illustrative materials containing recommendations formulated with different rating systems.

Discussion groups were moderated by members of the research team who had previous experience in that field, together with the collaboration of an observer, who collected field notes on anything that might be of interest for subsequent data analysis. No group interviews lasted more than ninety minutes. They were digitally audio-taped with the informants' consent and transcribed verbatim to obtain the final set of qualitative data for the analysis. The moderator initiated the discussion indirectly with an open-ended question (what comes to your mind when we speak about CGs?). During the session, the moderator assumed a minimally directive role, limiting intervention to prompting the group - if necessary - to address the topics in the script.

Data were analysed in a first step from a theoretical approach based on the grounded theory in which categories of analysis were defined. This was later complemented with a discursive sociological approach that analysed discursive positions, narrative configurations and semantic spaces. All the analyses were triangulated between several members of the research team, and discussed with those in charge of the moderation and observation in each group [26].

## Results

Physicians participating in the discussion groups showed that their perception of CGs was based on two main constructs related to their knowledge of CGs (such as those aspects determining the attitude of professionals toward the theoretical concept of CGs) and the possible usefulness of CGs (pragmatic characteristics). Both discursive constructs were involved in the following categories that shaped the participants' attitudes toward CGs and were also determinants of their ***utilization*** (implementation of the CPG in the setting of daily practice): ***confidence*** (position of trust that professionals confer to the theoretical information found in the CPG), ***access and dissemination*** (health professionals' modes of contact with CGs and their recommendations), ***format*** (aspects related to the way information is presented), ***recommendations*** (CGs conclusions as indications aimed at directing clinical practice and decision-making), and ***levels of scientific evidence*** (conceptual criteria of classification of the recommendations obtained from studies based on clinical practice).

### Theoretical conception and applicability to practice (Table 2)

The observed attitudes that define the knowledge construct that physicians have regarding CGs are based on a series of intellectual constructions on the characteristics of these documents, or the concepts supporting them. The main point in common when it comes to defining a CG, regardless of the methodological knowledge possessed by participants in the discussion groups in this respect, was that of the homogenizing function of CGs, which helps to standardize the practice, to organize assistance and, ultimately, to assist in clinical decision-making, assigning them an instrumental character (table 2).

**Table 2:** Knowledge of CGs (quotes).

**Practice Standardization**
*“(…) that's precisely what they are, some guidelines on how to proceed with our job”*
*“It's about homogenizing patient care to avoid uncertainties as to the steps to follow. It also includes patient involvement and the information we provide them”*
**Guidelines as Tools**
*“A tool with a summary of the existing evidence about how to manage particular conditions, in particular clinical situations, and also conditions about which there is a lack of evidence”*
*"I strongly believe it is about consensus and help in particular moments”*
*“Positive (…) I totally agree with you”*
*“(…) I'm pleased when I hear of clinical practice guidelines, I think they help at first with my decision-making”*
*“I believe we all agree that they are useful and also convincing”*
*“Of course, guides are useful but what people think of them is… they prefer protocols”*
*“I think they're important. It's important that there are people behind them, who have worked on them, who have developed them. And then it's important our work can be based on them”.*
*“The term ‘clinical practice guidelines’ seems to me more a question of aid in decision-making”*
*“(…) I believe we all agree that they are useful and also convincing. There has to be a change of mindset, knowledge, dissemination, working habits. Then we'll have a remarkable task before us if we are to work with guidelines.”*
**Barriers**
*“But not all patients may be included in the guidelines from there. Perhaps each case is different,…”*
*"They're cumbersome. They're so complete that you get lost in the attempt. The (…) guidelines were, literally, a weighty tome. (…) What can I say? Which are the positive aspects? You've got all these to choose from. All right, but which is the best one? That's not clear either…”*
*“(…) applicability is not sufficiently considered… That's why the implementation of the guidelines is often low. And I think this is so because not enough attention is paid to the setting where a given guideline is going to be applied”*
**Passive versus active attitude**
*“… professionals should start accepting their existence, and that they should be used as a reference, a guide for their work, their updating, their… studying to have updated knowledge and to know how to use them each time”*
*“What we want: to have the Ministry of Health come and say: ‘The hypertension guide is the one followed by the Spanish Society of Cardiology’”*
**Implementation**
*“There must be an institution that makes searching, systematization and gathering of data easier, an institution which presents you the data so that you are entitled to say: This is right, this is wrong’”*
*“What's very clear is that any guide, no matter how good they may be, has little chance of success if they are not disseminated, discussed locally, if adherence to the guides and the results of their follow-up are not monitored, …”*

Participants easily identified the theoretical concepts that define CGs, showing a positive perception of medical professionals toward CGs as tools based either on scientific literature, or on a consensus that should homogenize the clinical practice (table 2). We observed a disagreement between physicians' positive perception regarding CGs at a theoretical level and their applicability in practice. This disagreement stemmed from the identification of a series of barriers that limit this positive perception. Such barriers mainly corresponded to CGs that did not establish a process offering a clear solution and CGs that were not individually applicable to patients. However, they also corresponded to the vast quantity of information they gather, which hinders their comprehension and management in daily practice (table 2). Data show the link between theoretical knowledge on CGs (which defines its perception) and their practical knowledge and, ultimately, the possible use that can be made of them. Similarly, training was regarded as a facilitator of knowledge and use of CGs.

### Usefulness, utilization and implementation of the recommendations (Table 3)

As to the attitudes towards CGs use, two differentiated behavioural patterns or demands were observed. First, there was the motivation of professionals to carry out an active search of documents that could facilitate their clinical practice. Second, professionals appeared to have a passive attitude, expecting the workplace organization (planners, managers, etc) to facilitate access (table 3).

**Table 3:** Use of CGs (quotes).

**Uncertainty**
*“The evidence -be it a guide or in guide format- should be adapted to our context, and it should ease and reduce any uncertainty.”*
**Resolution of common problems versus less common problems**
*“Among the conditions, we should have faster access to those that are more common and discuss the less frequent ones in special sessions or as special cases…”*
*“… chronic diseases, hypertension, … you read the guide mainly to get acquainted with the way things are going in the field …”*
*“… I feel that the clinical practice guidelines are precisely most useful in the case of those conditions which I'm not used to handling on a day-to-day basis”*
*“… I have consulted them exceptionally, for particular cases where a decision has to be made and there's no one there to tell you what you should do…”*
**Barriers**
*“I believe they should be adapted to your own hospital, your own reality. Suppose the case of a liver whose only choice is a transplant. Well, it's obvious that not all contexts will provide the necessary facilities to perform it”.*
*“So then, whose judgment are we to follow? Don't you think so? Sometimes you don't know to which extent they're… which one is the most reliable or which one is the most applicable.”*
*“To control a given condition you have to revise eight, nine -at best- clinical practice guidelines [laughter]. Many of them, based on the same scientific evidence, most of them. And with often conflicting recommendations.”*
*“Do you have time to do evidence-based medicine in your office? Well, honestly, I don't.”*
**Knowledge**
*“… we should be given a pre-established time from our working hours for training”*
*“… our organization has not been much concerned with introducing them in our working hours”*
*“I refer to the institution in the sense that they should get involved in facilitating their use.”*
*“In my case, I've used them but there's a limit to it since the rest is oriented to the hospital, to hospital issues. I do not have the same access. That belongs to another level.”*
**Confidence**
*“I think that the guides are useful only for very well established matters (…) those who design the guides are people who are not currently involved in health care practice. They are often people who take part in committees. I know a few committees and they're people who have never seen a patient, or it's been long since they last saw one. So they sometimes beat about the bush while the problems we face in the practice are never resolved.”*
*“But they get unreliable when the pharmaceutical industry is behind. And this becomes a barrier.”*
*“Certainly, and despite all this, the pharmaceutical industry (…) exercises pressure and informs us much more than the official institutions preparing the clinical practice guidelines, who theoretically, and pragmatically, are less biased.” ZgzMHCon-H3*
*“To know the conflicts and interests they may have. To know who sponsors them, who promotes them and then read them critically and systematically to figure out where the clinical practice guideline comes from (…)”*
**Formats**
*“It's like the asthma GEMA guideline, where you have the there is the big one and the pocket one. And you can download both from the society site, can't you? And you have the large one with all the evidence, all you have to do, and a small one where things are more or less summarized and are more applicable. And this is what is useful in the end, because if you have a weighty tome that can't be applied in the practice, they give you some recommendations, some guidelines which have been agreed upon, and this is much more practical.”*
*“Yes, it is very difficult to implement, because of the use of the language, what is stated is very artificial. And although you may want to use a given word for that, our mind, at least in my case, will not work.”*
*“(…) the language is important, isn't it? The way it is written will affect how much of it you can actually get (…) I mean, the language has to be straightforward, including a recommendation which is in fact a recommendation.”*
*“I believe that they should treat this more seriously, use scientific language which is intelligible for everyone”*
*“Or where problems were standardized with algorithms that are easy to grasp, with their grade of recommendation and useful evidence, and this can be presented in electronic format to be used daily”.*
*“To implement it you have to know it first, and sometimes knowing it is to be up-to-date (…).”*
*“A clinical practice guideline (…) has to have implemented in its own updating mechanism.”*
*“(…) in fact, the clinical practice guidelines are a tremendously useful instrument to be up-to-date and in agreement with evidence-based medicine.”*

CGs use in daily practice implies a process of implementation, involving dissemination and training in these documents. These two meanings constitute facilitating elements that take part in a positive feedback process, given that a correct dissemination and training contribute to a better knowledge and use of CGs (table 3).

The perceived usefulness of CGs was identified as the main category filling the gap between theoretical knowledge and their use in the clinical practice. Physicians who participated in the discussion groups had a positive opinion about CGs as these tools can provide standards from which they can make the best possible decision when facing situations of uncertainty (table 3), although they were not recognized as resolution tools for all the problems they have to face in clinical practice.

As for the CGs aim of reducing the variability in clinical practice, two opposing attitudes have been identified among participants in the discussion groups. On the one hand, CGs are perceived as useful tools for a correct approach of the most frequent conditions, and on the other hand, they are also perceived as assistance tools to address less common health issues of a lesser complexity (table 3).

Availability of several formats of a CG is identified as another possible determinant of its use. Implementation and use of CGs could be facilitated by having a summarized version with concise information on the most important recommendations for clinical practice, submitting written recommendations in plain language with no ambiguities, and gaining access to an electronic format that allows for frequent updating (table 3).

Another determinant in the use of CGs was confidence. Although the development and formulation process of recommendations is perceived as an important factor, participants in the discussion groups ascribed much more importance to those who develop and endorse the CGs. The fact that CGs developers can be expert methodologists or professionals not involved in clinical practice generates some rejection and limits their implementation. A factor that contributed most to the confidence perceived by health care professionals was authorship of recommendations, which could lead to a positive or negative attitude toward the use of a given CG. The involvement of the pharmaceutical industry in the development of CGs generates a certain attitude of suspicion, and even rejection to the recommendations included in CGs developed or financed by the same company (table 3).

Certain aspects related to the usefulness of CGs create barriers to their use. One barrier is the inability of CGs to adapt or take into account the characteristics of the particular setting in which their recommendations are intended to be used, thus limiting its applicability. Their use is also limited by the availability of several CGs to address a single health problem; this makes it difficult to assimilate their contents, and recommendations may even diverge. Another point to consider is that updating of CGs may not keep up with scientific advances in certain pathologies, possibly triggering scepticism toward some of these documents (table 3).

### Levels of evidence and recommendations (Table 4)

The collected data show that the levels of scientific evidence and the recommendations are a common element between the two main categories built upon discourse analysis (knowledge and use of CGs), being located in a macro-discourse level. They appear in the theoretical plane (as one of the basic components in the development of CGs), but also as a category that could guarantee credibility and confidence in CGs, thus facilitating their use, dissemination, and access.

CGs methodological sections, with information such as tables synthesizing the evidence that back up the recommendations, were highly regarded by participants, since they are perceived as indicators of strictness, responsibility and transparency. However, although the inclusion of these sections in CGs adds value, it does not necessarily mean that users know how to handle them, since some knowledge of research methodology is needed to this end (table 4).

**Table 4:** Levels of evidence/Strength of recommendation (quotes).

**Methods**
*“Personally, if I see a guideline showing which level of evidence corresponds to each recommendation I consider it more reliable than another which does not mention this.” To my mind, this gives strength to the recommendation, or the suggestion, which sometimes says ‘We recommend or we suggest based on the evidence strength of the studies’. Indeed, this gives me more confidence.”*
*“I believe that the non-specialist should get the information predigested, not everyone should be digesting things around, but at the same time you just can't present it as if they were dummies…”*
*“In any case, I think professionals do not look at the methodology.”*
**Proposals symbols numbers systems**
*“Yes, it is very difficult to implement, because of the use of the language, what is stated is very artificial. And although you may want to use a given word for that, our mind, at least in my case, will not work. On the other hand, what she says is right, I see A, 1A or A. And that gets into my head.”*
*“On the other hand, if you say ‘It is recommended’ and then you decide for A, you know that's the best thing to do. Then you go for it. On the other hand, if they say ‘It is recommended…’, they give you the choice to be open to other choices. A is that instead.”*
*“Because it's only natural that you don't know about A, or B, or that 1B is better than C, or that 1 is the same as 4, or that 4 is the best there is because it's the highest, and that you are not sure about it… I understand that many people may find 1 easier to grasp.”*
**Need for homogeneity**
*“No, I'd prefer the same system is used every time. Or the one that is as homogeneous as possible, because then you can compare options. Because if I look at a guide (…) I need all the same to be told what it is, right? That's what I want. But it'd be far easier for me if they always used the same classification system.”*
*”They're tremendously cumbersome to me. I forget about them every time I look at them, and if I don't have the table at my side, and all the various systems, well, then, I just can't do it.”*

Although the moderators of the groups tried to explore participants' perception of the different ways in which levels of evidence and the strength of the recommendations were graded, participants showed little interest in the grading models used. In general, participants showed a preference for systems based on numbers and letters to classify the evidence and grade the strength of the recommendations (table 4). The lack of standardized classification systems unifying the many suggestions to date is responsible for considerable confusion among users who must move from one CG to the other, and is regarded as an important barrier that may condition CGs use.

## Discussion

Our study identified two main constructs explaining how physicians perceive CGs, and how this perception impacts on the attitude towards them. On one hand, there is a conception at the level of knowledge of CGs (from a theoretical construction) and, on the other hand, there are a series of categories related to their usefulness (from a pragmatic construction). The categories identified in the texts from the discussion groups shape aspects that enable us to relate these two main constructs, acting both as facilitators in the implementation of this conceptual perception of CGs, and as barriers to their use.

Overall, health care professionals show a positive attitude toward CGs, regarding them as useful tools to standardize clinical practice and shared decision-making. However, a number of factors appear to hinder their practical use. These factors are related to aspects such as a limited knowledge and limited access to CGs, lack of confidence in developers, lack of manageable formats in daily clinical practice, and the absence of standardized classification systems of the levels of evidence and recommendations.

Some barriers to the use of CGs have also been identified around these two constructs. The main sceptical attitudes toward CGs are related, on one hand, to the often difficult applicability of the scientific literature that supports recommendations at the individual level [27]. On the other hand, our study shows that the questioning of CGs and their usefulness is also rooted in the distance that separates the theoretical content collected by CGs from their realization in the clinical practice. This is mainly due to a limited adaptation of CGs to their contexts of application.

Our study suggests that an important barrier to the use of CGs, and a factor of physicians' perception of their usefulness, is the disagreement between the theoretical content of CGs and the daily practice. The role played by health professionals as primary actors in decision-making in clinical practice can differ from the objectives of CGs [28]. This represents a barrier to the interest that professionals show towards them, since there is a conflict between the recommendations presented by a CG and physicians' needs and experiences. We also found in the literature the need to incorporate front-line clinicians in the development of recommendations to improve the representativeness of CGs [12], [14].

As in other studies [12], [27], [28], participants in our discussion groups expressed the need for commitment in the dissemination and implementation of CGs on the part of their organizational structures. Participants highlighted the need to involve healthcare professionals with expertise in the area covered by the guideline scope to avoid methodologically rigorous documents but with limited applicability. In our context, the influence of the institutions or companies promoting CGs, funding source, and its authors are vital to the confidence that health professionals place in a CG. In this regard, the fact that a CG can be backed up or financed by the pharmaceutical industry is perceived with fear given the risk that their content might be conditioned by profitable intentions [27]. These aspects are even more relevant to participants in the study than the rigor of methods that sustain recommendations.

Some of the barriers to the use of CGs have been associated with practical aspects in our study, as other authors have also pointed out [12]. They are realized as aspects such as the lack of time to read or evaluate these tools, and this usually implies a setback for an adequate knowledge of CGs.

Some participants in our study expressed the need to disseminate CGs better before their implementation [27]. This includes receiving adequate training about the CGs content and their implications for their practice. However, this need for training is in conflict with settings having a high workload pressure and the limited time available to this end.

Dissemination, form of access to CGs, and CGs format are the main determinants of their knowledge and use. Overall, the literature also identifies format as a facilitator for their use [14]. Our study has identified the brief and simple formats as the aspects most appreciated by professionals. Accessibility stands out as a new component that adds value to the format of a CG. In this regard, it is of note that our study reinforces the positive perceptions received by the electronic formats mentioned in previous studies [27].

Contrary to our expectations, the participants in the discussion groups did not devote much time discussing grading systems. This is due to the natural complexity of qualitative designs and specifically in group discussions. A researcher who moderates group discussions has less control over the data produced than in in-depth interviews or quantitative studies. Participants expressed that the lack of standardized classification systems could be responsible for considerable confusion among users and a potential barrier to the use of CGs. This topic clearly deserves further research. Other authors highlight the importance of standardizing these systems to find a balance between rigour and simplicity [29].

Our study highlights the involvement that CGs developer groups should have when developing CGs aimed at answering specific and relevant questions. They should count on all the actors implicated, and they should be explicit, consistent and strict in the process of development. Finally, it is important that CGs are accompanied by working tools that strengthen their utility, trying to facilitate their dissemination and applicability, and taking into account the need to adapt CG formats to the specific characteristics of the different user profiles.
